# Impact of Using Unedited CT-Based DIR-Propagated Autocontours on Online ART for Pancreatic SBRT

**DOI:** 10.3389/fonc.2022.910792

**Published:** 2022-06-08

**Authors:** Alba Magallon-Baro, Maaike T. W. Milder, Patrick V. Granton, Wilhelm den Toom, Joost J. Nuyttens, Mischa S. Hoogeman

**Affiliations:** Department of Radiotherapy, Erasmus MC Cancer Institute, University Medical Center Rotterdam, Rotterdam, Netherlands

**Keywords:** pancreas, SBRT, adaptive, replanning, autocontouring

## Abstract

**Purpose:**

To determine the dosimetric impact of using unedited autocontours in daily plan adaptation of patients with locally advanced pancreatic cancer (LAPC) treated with stereotactic body radiotherapy using tumor tracking.

**Materials and Methods:**

The study included 98 daily CT scans of 35 LAPC patients. All scans were manually contoured (MAN), and included the PTV and main organs-at-risk (OAR): stomach, duodenum and bowel. Precision and MIM deformable image registration (DIR) methods followed by contour propagation were used to generate autocontour sets on the daily CT scans. Autocontours remained unedited, and were compared to MAN on the whole organs and at 3, 1 and 0.5 cm from the PTV. Manual and autocontoured OAR were used to generate daily plans using the VOLO™ optimizer, and were compared to non-adapted plans. Resulting planned doses were compared based on PTV coverage and OAR dose-constraints.

**Results:**

Overall, both algorithms reported a high agreement between unclipped MAN and autocontours, but showed worse results when being evaluated on the clipped structures at 1 cm and 0.5 cm from the PTV. Replanning with unedited autocontours resulted in better OAR sparing than non-adapted plans for 95% and 84% plans optimized using Precision and MIM autocontours, respectively, and obeyed OAR constraints in 64% and 56% of replans.

**Conclusion:**

For the majority of fractions, manual correction of autocontours could be avoided or be limited to the region closest to the PTV. This practice could further reduce the overall timings of adaptive radiotherapy workflows for patients with LAPC.

## Introduction

Adaptive radiotherapy (ART) is a desired paradigm in radiation therapy. Its goal is to adjust the treatment plan to the patient anatomy of the day to compensate for anatomical changes (1, 2). An online ART workflow has to be time efficient as the patient awaits treatment (1, 3). In recent years, efforts have been focused on speeding up the ART process through fast treatment plan reoptimization techniques and through automatically segmenting anatomical structures in medical images (3–10). The latter aims to reduce delineation times, which in ART remains a crucial point since contouring has been traditionally performed manually by dedicated and trained staff (11).

Carcinomas located close to radiosensitive and mobile organs-at-risk (OAR), such as unresectable locally advanced pancreatic cancer (LAPC), are excellent candidates for ART (4, 8, 9, 12). LAPC is a dose-limited tumor type, whose dosage is often compromised to protect surrounding organs. To manage this limitation, stereotactic body radiotherapy (SBRT) has become a standard of care for LAPC, owing to its capability to deliver highly conformal doses with steep dose gradients (13–17). Nonetheless, due to day-to-day OAR mobility, unintended doses are received by OAR close to the tumor (3, 18). For that reason, ART is recently being explored for LAPC patients using systems such as the MRIdian (ViewRay, Oakwook Village, OH) (8, 9, 12, 19, 20), the Elekta Unity (Elekta AB, Stockholm, Sweden) (7, 9, 21), or the Ethos (Varian Medical Systems Inc, Palo Alto, CA) (22, 23).

In our clinic, LAPC patients are treated on the CyberKnife (CK) (Accuray Inc, Sunnyvale, USA) using real-time tracking (24, 25). The CK does not have an integrated 3D imaging system, but our institute has a unique CT-on-rails in the treatment room that allows daily imaging (26). Our previous work investigated the potential trade-offs of applying different fast and quasi-automated plan adaptation methods on the CK (6). Nonetheless, a major challenge remains in laborious daily organ delineation, i.e. contouring.

Automatic contouring methods may offer a solution and are often based on the propagation of contours from the planning (pCT) to the fraction CT (FxCT) through deformable image registration (DIR) (2–4, 7). The use of automatic algorithms not only speeds up this task, but could also offer consistency to limit intra- and inter-observed variations. However, due to poor soft tissue contrast in the abdominal area, autosegmented organ contours (i.e. autocontours) generally require further manually editing before being used for daily replanning purposes (3, 27). Within an ART framework, manual delineation is one of the most time-consuming steps, but is thought to be essential to guarantee the quality of the adapted treatment plan. The time required for delineation delays the start of radiation delivery, and allows for additional intra-fraction OAR motion to occur, which can devaluate further the adapted plan. For this reason, in this study we have explored if manual editing of daily contours can be avoided while replanning. We have investigated the impact of using unedited autocontours generated with two commercially DIR algorithms available in Precision^TP^ (Accuray Inc, Sunnyvale, USA) and in MIM (MIM Software Inc, Cleveland, USA). The value of replanning directly on unedited autocontours has been established by: (a) comparing resulting plans to replans obtained using manual contours in the optimization, and (b) comparing them to conventional non-adapted SBRT plans. In addition, we also quantified the geometric accuracy of both DIR algorithms, especially close to the target volume.

## Materials and Methods

### Patient Data

A total of 35 patients with pancreatic cancer were included in this study. All patients were diagnosed with inoperable nonmetastatic LAPC, and presented a stable disease after receiving 8 cycles of chemotherapy (FOLFIRINOX). They received subsequent hypofractionated SBRT treatment of 40 Gy in 5 fractions, prescribed to the 80% isodose line. Patients gave informed consent to be included in the LAPC-1 Phase II study, which was approved by the Institutional Review Board (ID: NL49643.078.14) in accordance with the recommendations of the Declaration of Helsinki.

The study protocol indicated that each patient received a planning CT (pCT) and 3 contrast-enhanced in-room daily scans under instructed end-expiration breath-hold prior to treatment delivery (FxCT). All scans were acquired after manually injecting intravenous contrast agent, and by immobilizing patients using a vacuum bag on the treatment couch. Patients were recommended to avoid food and drink intake 2 h before the treatment fraction. In total, 98 FxCT were collected in this cohort, since only 2 daily CTs were available for 7 out of 35 patients.

The pCTs were delineated by a radiation oncologist (with 10+ years of experience) following the RTOG guidelines on the abdominal region (28). The gross tumor volume (GTV) was expanded by 5 mm to generate the clinical target volume (CTV), which was subsequently expanded by 2 mm to create the planning target volume (PTV). Additionally, the main organs-at-risk (stomach, duodenum, bowel, kidneys and liver) were also manually contoured.

Patients were treated using the CyberKnife M6 system with synchrony respiratory motion tracking on pre-implanted gold fiducial markers (24, 25, 29). Each patient had a median of 3 fiducials in or around the pancreatic tumor. The clinical protocol stated that 95% of the PTV should receive 95% of the prescribed dose (i.e., 40 Gy/5 fx), although PTV underdosage was allowed to fulfill OAR constraints. The stomach, duodenum and bowel had a near-maximum dose constraint of V35 Gy < 0.5 cc. For the liver, dose-constraint was V20 Gy < 700 cc, for the kidneys, mean dose < 15 Gy and V15 Gy < 30%, and for the spinal cord, allowed max dose was < 27.5 Gy.

### Delineations on the Daily Scans

#### Baseline of Manual Contour Set

FxCTs were delineated by the same radiation oncologist that delineated the pCT scans. The GTV and PTV were rigidly transferred to FxCTs after applying a fiducial pre-match. Additional details regarding OAR delineations can be seen in (30).

#### Autocontour Sets

Contours from the pCT were propagated to FxCTs using the deformable image registration (DIR) algorithm available in both Precision^TP^ (version 2.0.1.1) and MIM (version 6.9.3). A summary of each DIR method is available in **Supplementary Materials (A)**, as well as the procedure followed for parameter selection in MIM DIR. Whereas MIM DIR settings could be tuned to optimize the resulting contours for our dataset, Precision DIR settings are fixed and cannot be modified. The autocontours (AUTO) obtained using Precision DIR (asPREC) and MIM DIR (asMIM) remained unedited.

#### Contour Sets Geometrical Comparison

Both autocontours sets (asPREC and asMIM) were geometrically compared to MAN through the Dice coefficient (DC) (which describes the overlapping ratio between two volumes), mean surface distance (MSD), Hausdorff distance (HD) (which describes the maximum distance between two contour surfaces) and volumetric difference (VOL_DIFF) between the automatic vs. manual contours. These 4 accuracy metrics complement each other by giving an indication of the volumetric error and the distance between the structures boundaries, as recommended in Sharp et al. (2) and AAPM TG-132 (31). All metrics were collected using an in-house algorithm. Most of these metrics present a skewed distribution, and hence, median and interquartile range (IQR) parameters describing the data spread between quartile 1 (Q1) and 3 (Q3) (i.e. the 25% and 75% percentiles in which the distribution lies), were collected for the subsequent comparison analysis.

MAN, asPREC and asMIM stomach, duodenum and bowel structures (the closest OAR to the target and mostly located within the high dose region), were clipped at 3, 1 and 0.5 cm from the PTV for geometrical comparison (4–6). The resulting asPREC and asMIM clipped organs were compared to MAN clipped structures by means of DC, MSD, HD and VOL_DIFF metrics.

Since the three gastrointestinal (GIO) organs (i.e. stomach, duodenum and bowel) have the same dose-constraints in the clinical protocol, a structure combining the three was created at each different scenario (whole and clipped GIO at 3, 1 and 0.5 cm). GIO structures were also compared using DC, MSD, HD and VOL_DIFF. No recommendations on a combined GIO structure are included in the clinical protocol. The GIO structure was only created to evaluate the geometrical similarity of the combined organs, while minimizing the effect of registration errors in the transition between organs (e.g. stomach to duodenum).

The minimum distance (MIN_DIST) from GTV and PTV to OARs and the overlapping volume (OVLP) of the expanded PTV (with 0.5 and 1 cm) with the OAR was also retrieved for MAN, asPREC and asMIM.

### Replanning on MAN, asPREC and asMIM Contours

Treatment plans were optimized using the VOLO™ optimizer in Precision^TP^ (v2.0.1.1). As detailed in (6), a fast patient-specific template, including all clinically optimal cost functions used in the pCT, was generated. These fast templates reproduced the delivered clinical plans, while using a reduced number of nodes and OAR clipped at 3 cm from the PTV. These parameter combinations significantly reduced plan optimization times (6).

**Figure 1:**
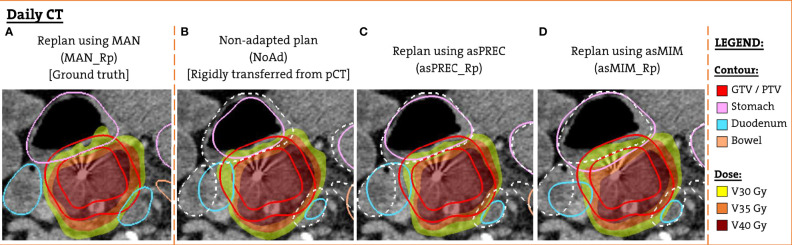
Example patient FxCT scan with the different structure set and dose distribution used for the dosimetric evaluation. **(A)** Replanned dose optimized using manual contours (ground truth). **(B)** Non-adapted dose with planning anatomy rigidly transferred from the pCT (solid lines). **(C)** Replanned dose optimized using contours obtained with Precision DIR (solid lines). **(D)** Replanned dose using contours from MIM DIR (solid lines). For **(B–D)** manual contours are also overlaid (dashed white lines).

The patient-specific templates were used to perform an automated full inverse planning on the pCT. These planning doses were rigidly transferred to FxCTs to evaluate non-adapted (NoAd) doses. We transferred the dose to the FxCT rather than recalculating it, as in our previous work (6) we saw clinically irrelevant dose differences in the OAR and in the target volumes when comparing transferred and recalculated plans. Next, the template was used to perform a new automated full inverse planning on the FxCT to generate adapted plans using the clipped MAN, asPREC and asMIM at 3 cm. The resulting adapted plans are referenced hereafter as MAN_Rp, asPREC_Rp and asMIM_Rp, respectively. **Figure 1** shows an example patient with the 4 planned doses that were created and evaluated on the FxCT scan, as well as the contours used to optimize each different plan.

### Dosimetric Plan Comparison

The four resulting doses in the FxCT scans (NoAd, MAN_Rp, asPREC_Rp and asMIM_Rp) were compared based on coverage, mean and minimum doses of the GTV and PTV, and near-maximum dose constraints (V35 < 0.5 cc) and mean doses of the OAR. All four doses were evaluated on the daily MAN contours during the subsequent dosimetric analysis, although plan optimization had been done using the planning contours (as in NoAd) or autocontours (as in asPREC_Rp and asMIM_Rp). Median and interquartile range (IQR) of these parameters were abstracted, and were compared using a two-sided Wilcoxon signed rank test, with a statistically significance defined by a p-value of < 0.05.

The following plan comparisons were performed. Firstly, replanned doses (MAN_Rp, asPREC_Rp and asMIM_Rp) were compared to non-adapted doses (NoAd) to determine the value of daily plan adaptation with respect to conventional planning. Secondly, replanned doses optimized using unedited autosegmented contours (asPREC_Rp and asMIM_Rp) were compared to replanned doses optimized using MAN, to determine the impact of inaccuracies in organ delineation on the replans.

To determine if autocontouring inaccuracies could be correlated with OAR constraints violations after replanning, the volumetric differences of auto vs. manual contours (i.e. VOL_DIFF) were compared between the fractions exceeding and the fractions not exceeding dose-constraints after replanning. VOL_DIFF was compared within different isotropic rings sets at different distances from the PTV: 0-1 vs 1-3 cm, 0-1.5 vs 1.5-3 cm, and 0-2 vs 2-3 cm. A Mann-Whitney test was performed to assess the differences between rings results. Statistical significance was set by a p-value < 0.05.

## Results

### Contour Sets Geometrical Comparison

**Figure 2:**
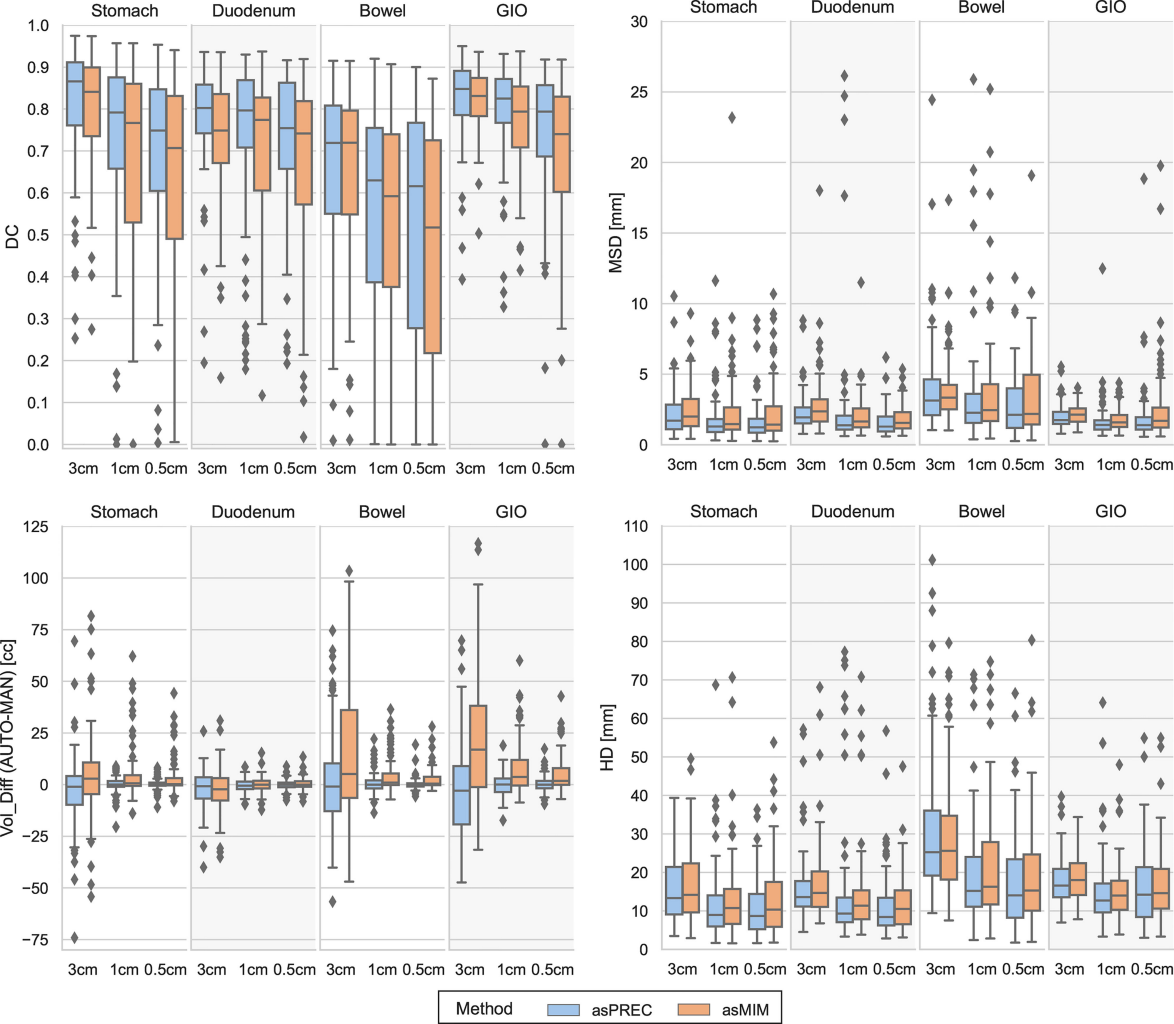
Boxplots showing the differences between Dice coefficient (DC) [top left], mean surface distance (MSD) [top right], volumetric difference between auto vs. manual contours (VOL_DIFF) [bottom left] and Hausdorff distance (HD) [bottom right] for structures autosegmented with Precision (asPREC in blue) and MIM (asMIM in orange). Each column of each subfigure distinguishes the boxplots on each structure (stomach, duodenum, bowel and GIO) and for each organ, distributions are separated for the clipped structures at 3, 1 and 0.5 cm from the PTV.

MAN, asPREC and asMIM contours were compared by means of DC, MSD, HD and VOL_DIFF on the whole (**Table B1**) and clipped OAR (**Figure 2** and **Table B2**), and by means of MIN_DIST and OVLP between target and OAR volumes (**Table 1**).

**Table 1:** Median and interquartile range (Q1, Q3) of the minimum distance (MIN_DIST) from GTV and PTV to OARs (stomach, duodenum and bowel), and the overlapping volume (OVLP) of the expanded PTV (at 0.5 and 1 cm) and OAR.

Metric	Method	Stomach	Duodenum	Bowel
	**MAN**	2.1 (-0.3, 6.9)	-0.3 (-0.8, 4.3)	9.4 (3.4, 15.0)
**MIN_DIST**	**asPREC**	2.3 (-0.6, 7.1)	0.0 (-1.7, 5.4)	9.7 (3.1, 20.8)
**GTV – OAR [mm]**	**asMIM**	1.2 (-1.5, 6.4)	-0.2 (-2.2, 4.6)	8.5 (0.5, 16.9)
	**(asPREC – MAN)**	-0.3 (-1.3, 1.3)	-0.5 (-1.4, 1.2)	0.4 (-1.5, 4.0)
	**(asMIM – MAN)**	-0.9 (-2.9, 0.4)	-0.9 (-2.1, 0.4)	-0.8 (-3.2, 2.0)
	**MAN**	-4.2 (-6.5, 0.4)	-6.4 (-7.4, -1.5)	3.1 (-2.6, 8.8)
**MIN_DIST**	**asPREC**	-4.0 (-6.9, 1.1)	-6.0 (-8.1, -1.0)	3.3 (-2.4, 14.2)
**PTV – OAR [mm]**	**asMIM**	-5.1 (-7.9, -0.1)	-6.5 (-8.6, -1.8)	2.3 (-5.5, 10.8)
	**(asPREC – MAN)**	-0.1 (-1.2, 1.3)	-0.4 (-1.2, 1.2)	0.6 (-1.3, 3.6)
	**(asMIM – MAN)**	-0.8 (-3.1, 0.6)	-0.8 (-2.2, 0.4)	-0.6 (-3.3, 1.9)
	**MAN**	3.4 (0.6, 8.2)	5.8 (1.5, 14.6)	0.0 (0.0, 1.6)
**OVLP**	**asPREC**	3.0 (0.5, 9.1)	5.6 (1.2, 16.5)	0.0 (0.0, 1.9)
**PTV_0.5cm - OAR [cc]**	**asMIM**	4.3 (0.8, 12.1)	6.6 (1.8, 17.3)	0.3 (0.0, 3.4)
	**(asPREC – MAN)**	0.0 (-0.4, 0.8)	0.0 (-1.2, 0.9)	0.0 (-0.4, 0.2)
	**(asMIM – MAN)**	0.2 (-0.3, 2.7)	0.0 (-0.9, 1.5)	0.0 (0.0, 1.4)
	**MAN**	9.5 (4.2, 18.7)	13.3 (4.4, 27.7)	1.7 (0.0, 6.7)
**OVLP**	**asPREC**	9.2 (2.9, 19.2)	12.2 (4.7, 29.6)	2.4 (0.0, 8.1)
**PTV_1cm - OAR [cc]**	**asMIM**	10.1 (4.4, 23.9)	13.4 (5.4, 29.7)	2.8 (0.0, 11.9)
	**(asPREC – MAN)**	0.0 (-1.0, 1.5)	-0.4 (-2.1, 1.5)	0.0 (-1.2, 1.7)
	**(asMIM – MAN)**	0.3 (-0.5, 3.8)	0.0 (-1.8, 1.9)	0.2 (-0.1, 4.4)

Results are presented for both manual (MAN), and autosegmented contours using Precision (asPREC) and MIM (asMIM), as well as the difference between auto and manual contours.

When evaluating the structures as a whole (**Table B1**), both algorithms reported high agreements between AUTO and MAN structures. A median (IQR: Q1, Q3) DC of 0.9 (0.9, 0.9), MSD of 2 (2, 3) mm, HD of 18 (15, 23) mm and VOL_DIFF of -1 (-16, 12) cc was observed for the combined GIO for asPREC, and a median DC of 0.9 (0.8, 0.9), MSD of 2 (2, 3) mm, HD of 19 (16, 23) mm and VOL_DIFF of 13 (-6, 27) cc for asMIM. The liver and kidneys were the organs reporting best results in both methods, and the bowel the worst, followed by the stomach and the duodenum.

When evaluating the clipped OAR at different distances from the PTV (**Figure 2** and **Table B2**), only the stomach, duodenum, bowel, and the combined GIO structure were considered. AUTO bowel contours were the structures showing less agreement with MAN bowels, followed by the duodenum and finally the stomach. Bowel contours reported the lowest DC, and larger MSD, HD and VOL_DIFF. The GIO structure generally outperformed individual organ measurements.

The DC in the 4 structures (i.e. stomach, duodenum, bowel and GIO) decreased closer to the PTV. Depending on the structure and method, DC ranged from 0.7 to 0.9 at 3 cm, and reduced to 0.5 to 0.8 at 0.5 cm distance from the PTV. The MSD showed little change at the 3 distances from the PTV, oscillating between 1 to 2 mm depending on the structure. The HD decreased for all structures when evaluated at 3 and 1 cm away of the PTV, reducing from a median of 18 to 13 mm in the GIO, but remained similar between 1 and 0.5 cm. Finally, the VOL_DIFF of AUTO vs. MAN reported similar volumes between MAN and asPREC. Conversely, asMIM showed positive differences compared to MAN ranging from 17 to 2 cc between 3 to 0.5 cm.

Generally, asPREC reported higher agreement with MAN than asMIM. As observed in **Figure 2** and **Table B2**, stomachs and bowels segmented with MIM were overestimated (i.e., positive VOL_DIFF), whereas with Precision both organs were slightly underestimated (i.e., negative VOL_DIFF). Both algorithms slightly underestimated the duodenum. Similar tendencies are observed in **Table 1**, in which asMIM reported smaller MIN_DIST to both GTV and PTV compared to MAN and asPREC, and also reported higher OVLP with the expanded PTV structure with autosegmented OAR.

### Dosimetric Comparison After Replanning

**Table 2** summarizes the dosimetric measurements performed in the non-adapted and adapted plans according to the different daily contours. After evaluating planned doses (NoAd) on MAN, 71% (70/98) of the plans resulted in OAR dose-constraint violations.

**Table 2:** Median and interquartile range (Q1, Q3) plan parameters of the replanned doses based on manual (MAN), and autosegmented contours using precision (asPREC) and MIM (asMIM) vs. non-adapted planned doses (NoAd).

Structure	Parameters	No adaptation (NoAd)	Replanning					
			MAN_Rp – NoAd	*ρ*	asPREC_Rp – NoAd	*ρ*	asMIM_Rp – NoAd	*ρ*
**PTV**	**Coverage (%)**	83.8 (78.0, 90.7)	-2.0 (-4.6, 0.1)	<.001	-2.7 (-4.5, -0.6)	<.001	-5.1 (-8.4, -2.6)	<.001
	**Dmean (Gy)**	43.1 (42.2, 44.1)	-0.5 (-1.0, 0.0)	<.001	-0.3 (-0.7, 0.0)	<.001	-0.7 (-1.2, -0.1)	<.001
	**Dmin (Gy)**	26.7 (25.5, 28.2)	-0.5 (-1.6, 0.3)	<.001	-0.7 (-1.4, 0.1)	<.001	-0.5 (-1.5, 0.2)	<.001
**GTV**	**Coverage (%)**	95.7 (91.1, 99.0)	-0.1 (-2.1, 0.6)	0.02	-0.4 (-1.9, 0.1)	<.001	-1.6 (-5.2, 0.0)	<.001
	**Dmean (Gy)**	45.8 (45.0, 46.5)	-0.5 (-1.0, 0.0)	<.001	-0.1 (-0.7, 0.2)	<.001	-0.5 (-1.2, 0.1)	<.001
**Stomach**	**V35 Gy (cc)**	0.2 (0.0, 0.8)	-0.2 (-0.8, 0.0)	<.001	-0.1 (-0.7, 0.0)	<.001	-0.1 (-0.4, 0.0)	<.001
	**Dmean (Gy)**	5.4 (3.3, 7.4)	-0.1 (-0.5, 0.5)	NS	-0.0 (-0.5, 0.5)	NS	-0.1 (-0.5, 0.4)	NS
**Duodenum**	**V35 Gy (cc)**	0.5 (0.1, 1.2)	-0.4 (-1.0, 0.0)	<.001	-0.2 (-0.7, 0.0)	<.001	-0.2 (-0.5, 0.0)	<.001
	**Dmean (Gy)**	9.7 (5.7, 12.7)	-0.3 (-1.1, 0.3)	<.001	-0.4 (-1.0, -0.1)	<.001	-0.4 (-0.9, 0.1)	<.001
**Bowel**	**V35 Gy (cc)**	0.0 (0.0, 0.3)	0.0 (-0.3, 0.0)	<.001	0.0 (-0.1, 0.0)	<.001	0.0 (-0.1, 0.0)	<.001
	**Dmean (Gy)**	1.9 (1.3, 2.6)	-0.1 (-0.3, 0.1)	<.001	-0.2 (-0.3, 0.0)	<.001	-0.2 (-0.3, 0.0)	<.001

Statistically not significant (NS) for p > 0.05.

Replanning based on MAN, asPREC and asMIM using a patient template resulted in plans satisfying OAR constraints (evaluated using MAN) for 93% (91/98), 64% (63/98) and 56% (55/98) of the fractions. Nonetheless, the V35Gy in unedited AUTO OARs was significantly lower in all organs compared to non-adapted plans for both asPREC and asMIM. Compared to NoAd plans, replanned doses on daily adapted contours (MAN, asPREC or asMIM) improved V35Gy in all OAR for 100% (98/98), 95% (93/98) and 84% (82/98) of the fractions. Using asPREC, the 5 fractions performing worse than NoAd occurred in 4 patients. Similarly, using asMIM, the 16 fractions performing worse than NoAD occurred in 14 patients. Median PTV coverage reduced by 2%, 2.7% and 5.1% compared to NoAD plans after replanning with MAN, asPREC and asMIM, respectively.

**Table 3** summarizes the differences between replanning using MAN vs. replanning using AUTO. V35Gy is significantly higher for the stomach and duodenum in plans based on autocontours compared to those based on MAN contours. This effect does not occur in the case of the bowel. **Table 3** also shows that the PTV coverage decreased when using AUTO. This result was not significant when replanning using asPREC, but was significant when using asMIM.

**Table 3:** Median and interquartile range (Q1, Q3) plan parameters of the replanned doses based on autosegmented contours using precision (asPREC) and MIM (asMIM) vs. replanned doses based on manual contours (MAN).

Structure	Parameters	Replanning				
		MAN_Rp	asPREC_Rp – MAN_Rp	*ρ*	asMIM_Rp – MAN_Rp	*ρ*
**PTV**	**Coverage (%)**	82.5 (75.1, 88.7)	-0.5 (-3.5, 1.6)	NS	-2.7 (-7.4, 0.2)	<.001
	**Dmean (Gy)**	42.5 (41.5, 43.7)	0.0 (-0.3, 0.6)	NS	-0.2 (-1.0, 0.4)	0.04
	**Dmin (Gy)**	26.4 (24.6, 28.0)	0.0 (-0.8, 0.8)	NS	0.0 (-1.1, 1.0)	NS
**GTV**	**Coverage (%)**	95.6 (90.7, 98.9)	-0.1 (-1.9, 1.1)	NS	-1.6 (-4.0, 0.0)	<.001
	**Dmean (Gy)**	45.3 (44.2, 46.1)	0.3 (-0.2, 0.9)	.001	0.0 (-0.8, 0.6)	NS
**Stomach**	**V35 Gy (cc)**	0.0 (0.0, 0.0)	0.0 (0.0, 0.1)	<.001	0.0 (0.0, 0.3)	<.001
	**Dmean (Gy)**	5.2 (3.2, 7.5)	0.0 (-0.3, 0.4)	NS	0.0 (-0.4, 0.5)	NS
**Duodenum**	**V35 Gy (cc)**	0.0 (0.0, 0.0)	0.1 (0.0, 0.4)	<.001	0.0 (0.0, 0.4)	<.001
	**Dmean (Gy)**	9.5 (4.9, 12.1)	-0.2 (-0.6, 0.2)	.02	-0.1 (-0.4, 0.3)	NS
**Bowel**	**V35 Gy (cc)**	0.0 (0.0, 0.0)	0.0 (0.0, 0.0)	NS	0.0 (0.0, 0.0)	NS
	**Dmean (Gy)**	1.8 (1.0, 2.6)	0.0 (-0.1, 0.1)	NS	0.0 (-0.2, 0.1)	.01

Statistically not significant (NS) for p > 0.05.

**Figure 3:**
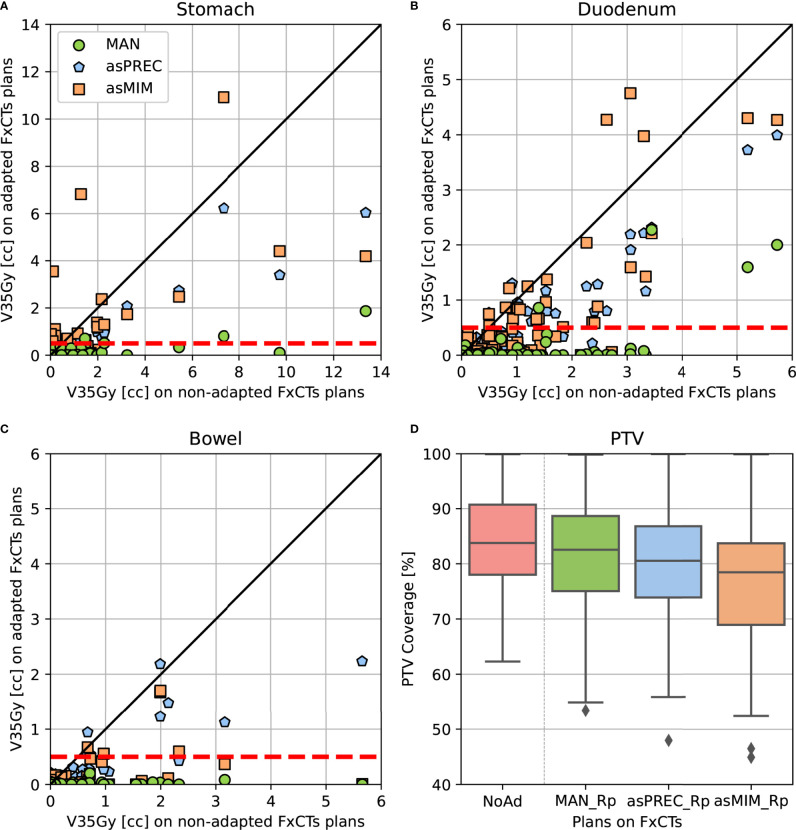
Pair-point comparison of OAR V35Gy parameter on non-adapted vs. adapted plans using manual and autosegmented contours with Precision (asPREC) and MIM (asMIM) on the stomach **(A)**, duodenum **(B)**, bowel **(C)**. Dashed lines depict OAR dose-constraints (V35Gy < 0.5 cc). In **(D)**, PTV coverage boxplot comparison of non-adapted (NoAd – red) vs. replanned doses: MAN_Rp (green), asPREC_Rp (blue) and asMIM_Rp (orange).

**Figure 3** shows the dosimetric parameters of adapted plans based on MAN, asPREC or asMIM vs. non-adapted plans. Dots located under the unity line (in diagonal) represent the dose distributions that improved compared to non-adapted plans. Similarly, dots located under the horizontal dashed red line at 0.5 cc on the y-axis represent the amount of adapted dose distributions that fulfilled the dose-constraints after adapting the plans using the three different contours sets. **Figure 3** visually presents the results from **Tables 2**, **3**: most plans fulfill the dose-constraints for the three organs after replanning at the cost of PTV coverage.

The correlation between autocontontour geometrical errors (assessed using VOL_DIFF of AUTO vs. MAN contours) and OAR violations (i.e., V35 Gy > 0.5 cc) were reported to be significant on all OAR within the ring of 0 to 1.5 cm from the PTV and not significant within the ring from 1.5 to 3 cm (**Table 4**). Other ring combinations results can be found in **Supl.Mat** (**Table B3**), but reported similar tendencies to **Table 4**. In short, large OAR autosegmentation inaccuracies (i.e., showing negative VOL_DIFF) occurring close to the PTV, appeared to be correlated with OAR violations after replanning. This correlation disappeared for large geometrical differences occurring at larger distances (i.e., within 1.5–3 cm ring from the PTV). **Tables 4** and **B3** suggest that recontouring efforts should primarily be addressed to OAR volumes close to the PTV, as this effort already solves most dose-constraint violations when replanning while minimizing the editing time involved.

**Table 4:** Median and interquartile range (Q1, Q3) of the volumetric difference of auto and manual contours in fractions violating and non-violating dose-constraints (V35Gy > 0.5cc) in the stomach, duodenum and bowel after replanning using precision (asPREC) and MIM (asMIM) autocontours.

Structure	Method	Distance to PTV	VOL_DIFF (AUTO – MAN) [cc]		
			Do not violate(V35 < 0.5 cc)	Violate(V35 > 0.5 cc)	*ρ*
**Stomach**	**asPREC**	**Ring 0 – 1.5 cm**	0.3 (-1.6, 2.0)	-10.9 (-13.6, -3.1)	.002
		**Ring 1.5 – 3 cm**	-1.9 (-7.2, 2.3)	-17.9 (-25.6, 3.4)	NS
	**asMIM**	**Ring 0 – 1.5 cm**	1.7 (-0.0, 6.2)	-6.2 (-10.2, 1.1)	<.001
		**Ring 1.5 – 3 cm**	1.0 (-4.2, 4.6)	-1.0 (-11.7, 8.1)	NS
**Duodenum**	**asPREC**	**Ring 0 – 1.5 cm**	0.2 (-2.3, 2.8)	-2.9 (-6.1, -1.1)	.001
		**Ring 1.5 – 3 cm**	-0.1 (-5.6, 1.7)	0.2 (-2.2, 4.8)	NS
	**asMIM**	**Ring 0 – 1.5 cm**	0.5 (-2.2, 2.7)	-3.0 (-7.4, 0.5)	.007
		**Ring 1.5 – 3 cm**	-0.4 (-7.1, 2.1)	0.3 (-6.7, 2.3)	NS
**Bowel**	**asPREC**	**Ring 0 – 1.5 cm**	0.5 (-1.5, 6.4)	-7.8 (-11.9, -4.9)	<.001
		**Ring 1.5 – 3 cm**	0.5 (-10.7, 8.2)	-6.0 (-10.1, -2.8)	NS
	**asMIM**	**Ring 0 – 1.5 cm**	1.0 (-0.8, 12.0)	-6.2 (-6.6, -3.4)	.017
		**Ring 1.5 – 3 cm**	6.6 (-3.7, 27.9)	2.5 (1.3, 4.5)	NS

Results are presented for the contour evaluated in the ring from 0 to 1.5 cm from the PTV vs. the ring from 1.5 to 3 cm from the PTV. Statistically not significant (NS) for p > 0.05.

## Discussion

Treatments using ART, especially online adaptive replanning, heavily rely on autosegmentation for a speedy and efficient workflow. However, current autosegmentation methods generally lack accuracy in the abdominal region and need to be followed by time and labor-intensive manual contour correction. In this study, we have quantified autocontouring quality of two commercially available software tools in the upper abdomen, and assessed the use of the resulting contours without further editing in daily replanning. Replanning with unedited contours resulted in better OAR sparing than non-adapted plans in 95% and 84% of plans optimized using Precision and MIM autocontours, respectively. For a large proportion of these fractions, resulting replanned doses stayed within OAR constraints (64% of plans when using Precision DIR, and 56% when using MIM DIR). Although autosegmentation inaccuracies can be located all over the OARs, the errors located closer to the PTV structure have the largest impact on OAR doses when replanning. These results suggest that manual editing of autosegmented OAR can be avoided in many fractions, but if applied, it can be limited to the region closest to the PTV to reduce the overall time of the ART workflow when treating patients with LAPC. Our research suggests that a cut-off limit of 1.5 cm could be sufficient, but an exact cut-off point requires further research and will be treatment protocol dependent.

A similar study was recently published using unedited contours for daily online ART in prostate patients using the Ethos system (32). In this study, the authors evaluated the gain of adapted plans with unedited contours vs. non-adapted plans. They report that 96% of their fractions would have required manual editing of the generated contours, but that 100% of the fractions achieved higher CTV coverage based on autocontours than using non-adapted plans. Similar to our work, the authors show that autocontouring methods are still inaccurate and require manual editing, but they also show that replanning on unedited contours is already beneficial compared to treating patient with non-adapted plans.

The added value of our work is that we also evaluated the dosimetric differences between adapted plans using manually corrected contours vs. using autocontours, hence, we also measured the potential gain in plan quality if autocontours are edited before replanning.

Regarding the geometrical analysis performed in our data, as expected, there were differences between manual and autocontours in the low and high dose region (within 3 cm from the PTV). Dice coefficient degraded when getting closer to the PTV. This is in part a natural expectation from this metric, as reports the overlapping ratio between 2 structures. The smaller the evaluated volumes, the more impact segmentation inaccuracies have. The Hausdorff distance measurement, reporting the maximum distance between 2 volumes, remains constant at different distances from the PTV, what reassures that there are relevant inaccuracies occurring close to the tumor.

Generally, contours propagated by Precision DIR showed a slightly higher agreement with manual contours than with MIM DIR, which tended to overestimate OARs (**Figure 2**, **Table B2**), and get closer to the tumor (**Table 1**). Consequently, asMIM_Rp dose distributions more often exceeded dose-constraints and lost more PTV coverage than asPREC_Rp. This difference between autocontour quality might be because Precision DIR optimizes the deformation vector field using localized patches within the image instead of the global image as done by MIM DIR (33–35) (see **Supl.Mat-A**).

Daily recontouring has traditionally relied on intra-patient contour propagation (as in this study) or atlas-based methods also using DIR (2, 3). Alternative autosegmentation methods are described in the literature, including artificial intelligence (AI). AI-based methods have shown improved accuracy and efficiency compared to traditional methods while being computationally very fast (36, 37). Several studies have shown improvements in different treatment sites (e.g. head-and-neck (38–40), prostate (39, 41), rectum (42), whole body (43)). However, abdominal organs present additional challenges including strong interpatient variability, bowel loop displacements and hollow organs, which causes AI studies still report similar results to those achieved in our current study (10, 44–46). Additionally, all studies focus on reporting autosegmentation accuracy on whole organ structures, whereas our results suggest mainly the accuracy close to the target influences plan quality.

Regarding replanning, manually corrected contours achieved the best results in OAR sparing compared to non-adapted plans (100% FxCT). However, replanning directly on unedited structures also improved OAR sparing for the large majority of fractions: 95% (93/98 FxCT) for Precision, and 84% (82/98 FxCT) for MIM. The corresponding 5 and 16 fractions in which plans based on autocontours increased OAR dose compared to non-adapted plans belonged to 4 and 14 patients, respectively. When looking further into the cases in which this phenomenon occurred (see two example cases in **Figure C1 in Supl. Mat.**), we noticed that manual contours were closer to the PTV than autosegmented contours, resulting in large inaccuracies close to the PTV for AUTO. Replanning on the autosegmented contours results in large dose violations, as the manual contours lie in the high dose area. Nonetheless, this poses a relatively small dosimetric risk for the patient especially taking into account that we analyzed single fractions rather than the total treatment dose, in which the effect of dose violations occurring in one single fraction, as in the case for the majority of our reported violations, is likely to be reduced.

Although OAR dose decreased when using unedited contours, the number of fractions obeying OAR constraints reduced compared to plans based on corrected contours. Also, PTV coverage generally decreased in fractions needing replanning. This similarly occurred when using MAN or asPREC, and slightly more often when using asMIM. Mostly, this was explained due to daily OAR moving closer to the high dose region or an increased OAR overlap with the PTV.

Our proposed implementation of ART is based on CT images and uses commercially available software. Although we are still in process of clinically implementing online adaptive replanning, we have performed end-to-end tests to mimic a clinical workflow. A complete adaptive procedure can be completed within 45 min, excluding treatment delivery, with room for improvement in delineation time. Similar to other publications, depending on the treatment site, editing of the contours on the FxCT – even when limited to a distance of 3 cm from the PTV - can take up a considerable amount of time in the entire procedure (around 10 min (4, 22, 27, 47)). The time of our total procedure is however in line with procedures performed on the MR-Linac (9, 12, 27), but is considerably longer than an online workflow on the Ethos system (22, 23). An inherent advantage of CyberKnife treatments is the excellent intra-fraction, both respiratory and non-respiratory, motion tracking. Currently this is lacking in the MR-Unity and Ethos systems leading to a possible increase in target size. The MRIdian is compensating for intra-fraction respiratory motion by means of gating.

Another limitation of our work is that we have a relatively small cohort group for this study. A validation involving an independent dataset potentially from other institutes should be performed to verify the relevance of our findings in pancreatic cancer. Although MR-Linacs and the Ethos systems rely on different imaging modalities, we believe our results could be transferred to other systems. For instance, similar trends were already observed in the work of Moazzezi et al. about online ART using unedited contours in prostate patients using the Ethos system (32). However, the complexity of the procedure might increase as the amount of elements involved also increases, e.g. generating correct Hounsfield Units.

Finally, intrafraction OAR motion has not been accounted for in this study. In our clinic, we use Synchrony respiratory motion tracking to mitigate the effect of intrafraction motion of the target, of which the accuracy has been reported elsewhere (25). Generally, intrafraction OAR variations while tracking are expected to be smaller than interfraction variations. Replans based on unedited contours already correct for interfraction OAR variations and generally outperform non-adapted plans in this study. We believe intrafraction OAR variations will have a smaller impact on the replans.

In conclusion, autosegmentation methods applying contour propagation after DIR in the abdominal region result in contours requiring manual correction. However, replanning on the unedited daily contours generally resulted in higher organ sparing than treating with a conventional SBRT scheme. In the majority of fractions, it even resulted in plans obeying the tight OAR dose constraints of our clinical protocol. In a large number of fractions, manual editing of automatic contours could, therefore, be avoided or at least restricted to contour sections in close proximity to the PTV, reducing the time required for online adaptive treatments for pancreatic cancer patients.

## Data Availability Statement

The original contributions presented in the study are included in the article/**Supplementary Material**. Further inquiries can be directed to the corresponding author.

## Ethics Statement

The studies involving human participants were reviewed and approved by Institutional Review Board (ID: NL49643.078.14). The patients/participants provided their written informed consent to participate in this study.

## Author Contributions

AM-B: conducting main research, data interpretation and analysis, and writing manuscript. MM, PG, and MH: supervision of research, contributed to the study design and writing manuscript. WT: contributed to data collection. JN: PI of data collection. All authors contributed to the article and approved the submitted version.

## Funding

This work was in part funded by a research grant of Accuray Inc., Sunnyvale, USA. Erasmus MC Cancer Institute also has a research collaboration with Elekta AB, Stockholm, Sweden and Varian, Paolo Alto, USA. The funder was not involved in the study design, collection, analysis, interpretation of data, the writing of this article or the decision to submit it for publication.

## Conflict of Interest

All authors are employed by the Erasmus MC. MM and MH report serving as an advisory board member for Accuray during the conduct of the study.

## Publisher’s Note

All claims expressed in this article are solely those of the authors and do not necessarily represent those of their affiliated organizations, or those of the publisher, the editors and the reviewers. Any product that may be evaluated in this article, or claim that may be made by its manufacturer, is not guaranteed or endorsed by the publisher.
