# Supplementary material for: Impact of Using Unedited CT-Based DIR-Propagated Autocontours on Online ART for Pancreatic SBRT
Source: Front Oncol. 2022 Jun 8;12:910792. doi: 10.3389/fonc.2022.910792 (PMC9213731; doi:10.3389/fonc.2022.910792)
Supplement: Supplementary file 1 [file DataSheet_1.pdf]

Supplementary materials

A. Autocontoring methods

A.1. Precision contour set on the daily scans

The novel DIR algorithm available in Precision treatment planning sysptem applies a non-parametric non-rigid transformation to generate the deformation field. The transformation across the whole image is calculated by optimizing the normalized correlation coefficient in small patches of voxels using an iterative approach, with the patch size decreasing at each iteration. A smoothness operator is applied to regularize the deformation field during each iteration.

Additional details of Precision DIR algorithm can be found in the Accuray DIR white paper [1] and Gupta et. al [2].

A.2. MIM contour set on the daily scans

The intensity-based deformable registration method available in MIM software (version 6.9.3) was used for contour propagation. This is the default DIR algorithm to register images of the same modality, as suggested by the manufacturer. The DIR method applies a constrained free-form registration, aimed to globally minimize the intensity differences between both fixed and moving images. The global deformation field is determined using a regularized coarse-to-fine multi-resolution approach on a set of control points on the fixed image. These control points are used to find the corresponding target volume in the moving image, using a custom modified gradient descent optimization strategy.

Different DIR parameters available in MIM were tested on two scans of 6 patients to determine which combination of DIR settings resulted in autosegmented contours with closest resemblance to MAN contours. In particular, experiments evaluated two different MIM DIR algorithm versions (<MIM 6.6.0 and MIM 6.6.0+); different smoothness factors (i.e. 0.1, 0.5, 1.0, 5.0, 10.0) which controls the motion allowed to occur within the deformation field; enabled normalization, which scales the intensity of one image to the intensity of the other; dynamic regularization, which uses the uniformity of the image region to help guiding the registration; and finally, density replacement within air bubbles, by masking air and replacing their intensity values prior to DIR. To determine the best parameter combination, the resulting autosegmented structures were geometrically compared to the corresponding MAN through the Dice coefficient (DC), mean surface distance (MSD) and Hausdorff distance (HD) metrics.

The best parameter combination was used to perform DIR and contour propagation between FxCT and pCT scans for all patients in the database. The final selected parameters were smoothness factor of 0.5 combined with Dynamic regularization, as decided from the results summarized from Table A1 below.

Additional details of MIM DIR algorithm can be found in the MIM's VoxAlign Deformation Engine white paper [3].

Experiment	All 6 OAR (Stomach + Duodenum + Bowel + Liver + Kidneys)			GIO (Stomach + Duodenum + Bowel)		
	DC	MSD [mm]	HD [mm]	DC	MSD [mm]	HD [mm]
SF 0.1	0.9	2.3	17.0	0.8	3.1	18.9
SF 0.5	0.9	2.3	16.3	0.8	3.1	18.6
SF 0.5 + Dyn	0.9	2.3	15.9	0.8	3.1	18.0
SF 0.5 + Dyn + AirBub	0.9	2.3	16.3	0.8	3.1	18.6
SF 0.5 + Norm	0.9	2.5	16.5	0.8	3.5	19.5
SF 0.5 + Norm + Dyn	0.9	2.5	16.4	0.8	3.5	19.2
SF 0.5 (< v6.6.0)	0.9	2.5	18.7	0.8	3.3	21.7
SF 1.0	0.9	2.4	16.0	0.8	3.3	18.6
SF 5.0	0.8	3.5	18.3	0.7	4.2	21.9
SF 10.0	0.8	3.9	18.6	0.7	4.6	22.4

Table A1. Mean of the Dice coefficient (DC), mean surface distance (MSD) and Hausdorff distance (HD) of the autosegmented set of OAR using different parameter combinations in MIM software. Results are abstracted for the 6 OAR altogether (i.e. stomach, duodenum, bowel, liver and both kidneys), and the 3 main gastrointestinal OAR (GIO) altogether (i.e. stomach, duodenum, bowel). The parameter combination used for the experiments of the current study is highlighted in gray.

A.3. References in A

[1] Jordan P, Myronenko A, Gorczowski K, Foskey M, Holloway R, Maurer CR. Accuray deformable image registration: description and evaluation. White Pap Accuray Software, Accuray Precis 2017:1–8.

[2] Gupta V, Wang Y, Méndez Romero A, Myronenko A, Jordan P, Maurer C, et al. Fast and robust adaptation of organs-at-risk delineations from planning scans to match daily anatomy in pre-treatment scans for online-adaptive radiotherapy of abdominal tumors. Radiother Oncol 2018;127:332–8. https://doi.org/10.1016/j.radonc.2018.02.014.

[3] Piper JW, Richmond JH, Nelson AS. VoxAlign Deformation Engine ® Deformable Algorithms 2018.

B. Complementary Tables

Metric	Method	Stomach	Duodenum	Bowel	Liver	Kidney right	Kidney left	All (6 OAR)
DC	asPREC	0.9 (0.8, 0.9)	0.8 (0.7, 0.9)	0.8 (0.8, 0.9)	1 (1, 1)	1 (0.9, 1)	1 (0.9, 1)	**0.9 (0.9, 0.9)**
	asMIM	0.9 (0.8, 0.9)	0.8 (0.7, 0.8)	0.8 (0.8, 0.9)	1 (1, 1)	1 (0.9, 1)	0.9 (0.9, 1)	**0.9 (0.8, 0.9)**
MSD [mm]	asPREC	3 (2, 4)	2 (2, 3)	4 (3, 5)	1 (1, 2)	1 (1, 1)	1 (1, 1)	**2 (2, 3)**
	asMIM	3 (2, 4)	3 (2, 3)	5 (4, 5)	1 (1, 2)	1 (1, 1)	1 (1, 2)	**2 (2, 3)**
HD [mm]	asPREC	22 (16, 28)	16 (13, 20)	32 (25, 39)	18 (13, 23)	11 (7, 16)	10 (6, 14)	**18 (15, 23)**
	asMIM	21 (15, 28)	17 (13, 21)	33 (27, 39)	19 (14, 25)	11 (7, 16)	10 (6, 14)	**19 (16, 23)**
VOL_DIFF (MAN-AUTO) [cc]	asPREC	-25 (-79, 5)	-1 (-9, 7)	-7 (-93, 99)	21 (-1, 59)	0 (-4, 10)	5 (0, 13)	**-1 (-16, 12)**
	asMIM	-11 (-43, 19)	-4 (-14, 5)	88 (-25, 223)	0 (-42, 39)	-3 (-9, 5)	2 (-5, 10)	**13 (-6, 27)**

Table B1. Median and interquartile range (Q1, Q3) of the Dice coefficient (DC), mean surface distance (MSD), Hausdorff distance (HD) and volumetric difference (VOL_DIFF) of the autosegmented set of the all OAR using Precision (asPREC) and MIM (asMIM) algorithms. Results are abstracted for the whole structure of the stomach, duodenum, bowel, liver and both kidneys, as well as the overall average among these 6 OARs.

Metric	Distance from PTV	Method	Stomach	Duodenum	Bowel	GIO
DC	3 cm	asPREC	0.9 (0.8, 0.9)	0.8 (0.7, 0.9)	0.7 (0.6, 0.8)	0.8 (0.8, 0.9)
		asMIM	0.8 (0.7, 0.9)	0.7 (0.7, 0.8)	0.7 (0.5, 0.8)	0.8 (0.8, 0.9)
	1 cm	asPREC	0.8 (0.7, 0.9)	0.8 (0.7, 0.9)	0.6 (0.4, 0.8)	0.8 (0.8, 0.9)
		asMIM	0.8 (0.5, 0.9)	0.8 (0.6, 0.8)	0.6 (0.4, 0.7)	0.8 (0.7, 0.9)
	0.5 cm	asPREC	0.7 (0.6, 0.8)	0.8 (0.7, 0.9)	0.6 (0.3, 0.8)	0.8 (0.7, 0.9)
		asMIM	0.7 (0.5, 0.8)	0.7 (0.6, 0.8)	0.5 (0.2, 0.7)	0.7 (0.6, 0.8)
MSD [mm]	3 cm	asPREC	2 (1, 3)	2 (2, 3)	3 (2, 5)	2 (1, 2)
		asMIM	2 (1, 3)	2 (2, 3)	3 (3, 4)	2 (2, 3)
	1 cm	asPREC	1 (1, 2)	1 (1, 2)	2 (2, 4)	1 (1, 2)
		asMIM	1 (1, 3)	2 (1, 3)	2 (2, 4)	2 (1, 2)
	0.5 cm	asPREC	1 (1, 2)	1 (1, 2)	2 (1, 4)	1 (1, 2)
		asMIM	1 (1, 3)	2 (1, 2)	2 (1, 5)	2 (1, 3)
HD [mm]	3 cm	asPREC	13 (9, 21)	14 (11, 18)	25 (19, 36)	17 (14, 21)
		asMIM	14 (10, 22)	15 (11, 20)	26 (18, 35)	18 (14, 22)
	1 cm	asPREC	9 (6, 14)	9 (7, 13)	15 (11, 24)	13 (10, 17)
		asMIM	11 (7, 16)	11 (8, 15)	16 (12, 28)	14 (10, 18)
	0.5 cm	asPREC	9 (5, 14)	8 (6, 13)	14 (8, 23)	14 (8, 21)
		asMIM	10 (6, 18)	11 (7, 15)	15 (10, 25)	15 (11, 21)
VOL_DIFF (AUTO - MAN) [cc]	3 cm	asPREC	-1 (-10, 4)	-1 (-7, 4)	-1 (-13, 10)	-3 (-19, 9)
		asMIM	3 (-5, 11)	-2 (-8, 3)	5 (-6, 36)	17 (-1, 38)
	1 cm	asPREC	0 (-1, 2)	0 (-2, 2)	0 (-2, 2)	0 (-4, 3)
		asMIM	1 (-1, 5)	0 (-2, 2)	1 (0, 5)	4 (0, 12)
	0.5 cm	asPREC	0 (-1, 1)	0 (-1, 1)	0 (-1, 1)	0 (-2, 2)
		asMIM	0 (0, 3)	0 (-1, 2)	0 (0, 4)	2 (0, 8)

Table B2. Median and interquartile range (Q1, Q3) of the Dice coefficient (DC), mean surface distance (MSD), Hausdorff distance (HD) and volumetric differences of the automatic vs. manual contours (VOL_DIFF [AUTO-MAN]). Results are presented for the set of autocontours achieved using Precision (asPREC) and MIM (asMIM). Parameters were acquired on the stomach, duodenum, bowel and the gastrointestinal structure (GIO), which is the combined volume of the three previous OARs.

			VOL_DIFF (AUTO – MAN) [cc]				VOL_DIFF (AUTO – MAN) [cc]		
**Structure**	**Method**	**Distance to PTV**	**Do not violate**	**Violate**	***ρ***	**Distance to PTV**	**Do not violate**	**Violate**	***ρ***
**Stomach**	**asPREC**	**Ring 0–1**	0 (0, 1)	-6 (-8, -2)	.001	**Ring 0–2**	0 (-2, 2)	-16 (-18, -3)	.004
		**Ring 1–3**	-1 (-8, 2)	-22 (-30, 3)	NS	**Ring 2–3**	-1 (-6, 1)	-12 (-19, 3)	NS
	**asMIM**	**Ring 0–1**	1 (0, 4)	-5 (-6, 0)	<.001	**Ring 0–2**	1 (0, 8)	-5 (-14, 2)	.002
		**Ring 1–3**	1 (-3, 5)	-3 (-16, 8)	NS	**Ring 2–3**	0 (-3, 3)	0 (-7, 6)	NS
**Duodenum**	**asPREC**	**Ring 0–1**	0 (-1, 2)	-3 (-4, 0)	<.001	**Ring 0–2**	0 (-3, 2)	-2 (-7, 0)	.02
		**Ring 1–3**	0 (-6, 2)	0 (-4, 4)	NS	**Ring 2–3**	0 (-3, 1)	0 (-1, 4)	0.04
	**asMIM**	**Ring 0–1**	0 (-1, 2)	-2 (-5, 0)	.002	**Ring 0–2**	0 (-3, 3)	-2 (-9, 0)	.01
		**Ring 1–3**	-1 (-6, 2)	0 (-9, 2)	NS	**Ring 2–3**	0 (-5, 1)	0 (-3, 2)	NS
**Bowel**	**asPREC**	**Ring 0–1**	0 (0, 2)	-5 (-8, -3)	<.001	**Ring 0–2**	0 (-3, 7)	-9 (-15, -3)	<.001
		**Ring 1–3**	1 (-10, 12)	-8 (-12, -4)	.05	**Ring 2–3**	0 (-8, 6)	-3 (-9, 0)	NS
	**asMIM**	**Ring 0–1**	1 (0, 6)	-4 (-4, -3)	.02	**Ring 0–2**	3 (-2, 19)	-5 (-6, -2)	.04
		**Ring 1–3**	10 (-3, 34)	3 (2, 4)	NS	**Ring 2–3**	4 (-3, 17)	1 (-2, 4)	NS

Table B3. Median and interquartile range (Q1, Q3) of the volumetric difference of auto and manual contours in fractions exceeding (i.e. V35Gy > 0.5cc) or not dose-constraints (i.e. V35Gy < 0.5cc) in the stomach, duodenum and bowel after replanning using Precision (asPREC) and MIM (asMIM) autocontours. Results are presented for the contour evaluated in the ring from 0 to 1 (left) or 2 (right) cm from the PTV vs. the ring from 1 (left) or 2 (right) to 3 cm from the PTV. Statistically not significant (NS) for $p > 0.05$.

C. Complementary Figures

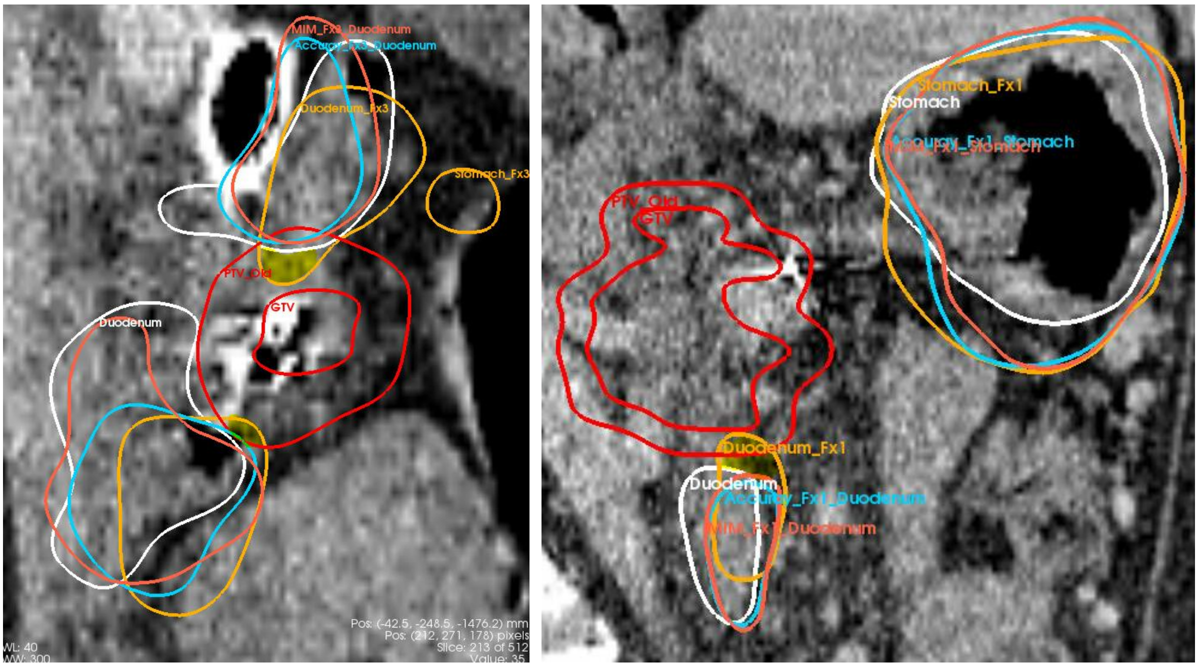

Figure C1. Two example patients in which adaptive plans were worse than non-adapted plans. See autocontours generated with Precision DIR (in blue) and from MIM DIR (in orange), which resemble more to the pCT anatomy (in white), rather than the FxCT manual contour (in yellow). Due to the inaccuracy of auto-contouring followed by dose optimization on the wrong volume, the voxels highlighted (in yellow) lead to a dose-constraint violation when evaluating the adapted plans on the ground truth FxCT manual contours.
